# Supplementary material for: SwsB Acts as a Muramic-δ-lactam Cyclase in Bacillus subtilis Spore Peptidoglycan Synthesis
Source: Biochemistry. 2025 Aug 28;64(18):3814–8. doi: 10.1021/acs.biochem.5c00449 (PMC12444981; doi:10.1021/acs.biochem.5c00449)
Supplement: Supplementary file 1 [file bi5c00449_si_001.pdf]

SUPPORTING INFORMATION

SwsB acts as a muramic-δ-lactam cyclase in *Bacillus subtilis* spore peptidoglycan synthesis

Madison E. Hopkins,[†] Zachary Yasinov,[†] Mark J. Fakler, Grace E. Wilde, Katherine G. Wetmore, and Michael A. Welsh*

Chemistry Department, Hamilton College, Clinton, New York, 13323, United States

[†] These authors contributed equally.
* Correspondence: mwelsh@hamilton.edu

METHODS

Materials. Unless indicated otherwise, all reagents and chemicals were purchased from Sigma-Aldrich and used without further purification. Oligonucleotide PCR primers were purchased from Integrated DNA Technologies. *B. subtilis* Lipid II was extracted and purified as reported.[1] *S. aureus* SgtB, *B. subtilis* CwlD, and PdaA variants (Table S1) were purified as previously described.[2,3] Mini-PROTEAN TGX gels (4-20%, Bio-Rad) were used for SDS-PAGE.

Bacterial Culture. The bacterial strains and plasmids used in this study are listed in Table S2. *E. coli* strains were grown at 37 °C with shaking in LB broth (Miller, Beckton Dickinson) or on agarized plates. Antibiotics were used at the following concentrations: ampicillin, 100 μg/mL; carbenicillin, 100 μg/mL; kanamycin, 50 μg/mL.

Cloning of *B. subtilis* SwsB. Plasmid pET24b was linearized by PCR using primers oMW47 and oMW104 (Table S3). The *swsB[K45-K319]* gene, which truncates a predicted N-terminal signal peptide and nonpolar α-helix, was amplified from *B. subtilis* PY79 genomic DNA using the primers oMW181 and oMW182. The DNA products were gel purified and joined by isothermal assembly using Gibson Assembly Master Mix (New England Biolabs) to produce plasmid pMW1295, which expresses *B. subtilis* SwsB[K45-K319] with a C-terminal His_6-tag. To generate a SwsB expression construct completely lacking the N-terminal domain, *swsB[E115-K319]* was amplified using primers oMW211 and oMW182 and joined with linearized pET24b as above.

Site-directed mutagenesis of *B. subtilis* SwsB and PdaA. Site-directed mutagenesis of SwsB and PdaA was conducted by amplifying the respective expression vectors with PCR primers encoding the mutation and circularizing the linear product using kinase, ligase, Dpn1 reaction mix (KLD, New England Biolabs). Primers used for each mutation are listed in Table S3.

Protein expression and purification – general protocol used for all proteins. *E. coli* C43(DE3) containing the appropriate plasmid was grown in 500 mL LB broth supplemented with kanamycin at 37 ºC with shaking until the OD600 was 0.5-0.6. The culture was cooled to 16 ºC before inducing protein expression with 500 μM isopropyl-β-D-thiolgalactopyranoside (IPTG) and shaking for 16 h. Cells were harvested by centrifugation (7,000 × g, 15 min, 4 ºC) and resuspended in 20 mL lysis buffer (50 mM HEPES pH 7.5, 400 mM NaCl). The cell suspension was supplemented with DNase (0.2 mg/mL) and phenylmethanesulfonyl fluoride (1 mM) and the cells lysed by passage through a cell homogenizer (EmulsiFlex-C5, Avestin) at ≥ 10,000 psi. Cell debris was pelleted by centrifugation (20,000 × *g*, 30 min, 4 ºC). The resulting supernatant was supplemented with 20 mM imidazole and then rocked with 0.5 mL Ni-NTA resin (Qiagen) for 45 min at 4 ºC. The resin was collected in a column by gravity flow and then washed twice with 5 mL wash buffer (50 mM HEPES pH 7.5, 400 mM NaCl, 40 mM imidazole). The protein was eluted in 10 mL elution buffer (50 mM HEPES pH 7.5, 400 mM NaCl, 200 mM imidazole) and concentrated by centrifugal filtration. The protein was then further purified by fast protein liquid chromatography (FPLC, AKTA Go, Cytiva) on a Superdex 200 Increase 10/300 column (Cytiva) in a running buffer consisting of 50 mM HEPES pH 7.5, 400 mM NaCl. Pooled elution fractions were concentrated by centrifugal filtration and the protein absorbance measured at 280 nm. The

predicted extinction coefficient of the protein (via ProtParam[4]) was used to estimate concentration. Proteins were diluted to 200 μM in running buffer with 10% glycerol (v/v), aliquoted, and stored at -80 ºC.

Purification of Metal-stripped Proteins. Proteins were expressed in *E. coli* C43(DE3) and purified by Ni-affinity pulldown at described above. The eluted protein was concentrated to 0.5-1 mL and dialyzed overnight in 1 L of 20 mM HEPES pH 7.5, 400 mM NaCl, 10 mM ethlylenediaminetetraacetic acid (EDTA). The protein was then further purified by FPLC as above in a running buffer of 50 mM HEPES pH 7.5, 400 mM NaCl, 10 mM EDTA. Proteins were diluted to 200 μM in this running buffer with 10% glycerol (v/v), aliquoted, and stored at -80 ºC.

Biochemical reactions – general conditions. Reaction conditions were adapted from a previously published protocol.[3] *B. subtilis* Lipid II was polymerized with SgtB, a monofunctional peptidoglycan glycosyltransferase from *Staphylococcus aureus*. Pooled polymerization reactions of up to 1 mL total volume were assembled under the following conditions: 50 mM HEPES, pH 7.5, 2 mM $CaCl_2$, 20 μM *B. subtilis* Lipid II, 0.2 μM SgtB, 10% DMSO (v/v). Reactions were incubated at room temperature for 30 min. *Bs*CwlD was added at 2 μM and the mixture incubated 1 h at room temperature, generating products enriched in peptide-cleaved MurNAc. When required, *Cd*PdaA was added at 2 μM for 10 min to generate polymer enriched in muramic acid (Mur) residues. To remove the enzymes, the entire reaction volume was passed over a 100 μL plug of settled Ni-NTA resin (Qiagen) by gravity flow twice. The eluate was then split into 50 μL aliquots. *Bs*PdaA or *Bs*SwsB were re-added at 2 μM and the reactions incubated at room temperature. Reactions were quenched by addition of 250 μL of methanol. Samples were then dried in a centrifugal evaporator and the residue resuspended in 50 μL of water.

Polymer reaction products were digested with mutanolysin from *Streptomyces globisporus* (Sigma Aldrich) to before LC-MS analysis. To 50 μL aliquots of reaction products, 8 U of mutanolysin was added and the reaction incubated at 37 ºC for 2 h with shaking. Aqueous sodium borohydride (10 mg/mL, 50 μL) was added and the reaction incubated for 30 min at room temperature. The solution pH was adjusted to ~4 by addition of 20% phosphoric acid (approximately 5 μL) and the reactions lyophilized to dryness overnight. The residue was dissolved in 25 μL of water and analyzed by LC-MS.

LC-MS Analysis of Reaction Products. LC-MS was conducted on a Thermo Scientific Vanquish HPLC in line with a Thermo LTQ XL ion trap mass spectrometer using electrospray ionization (spray voltage, 4500 V; spray temp, 300 ºC; capillary voltage, 46 V; capillary temp, 275 ºC) and operating in positive ion mode. Reaction products were separated on a Waters Cortecs T3 column (120 Å, 1.6 μm, 2.1 x 50 mm) equipped with a matching column guard using the following method: 0.6 mL/min eluent A (water/0.1% formic acid) for 2 min followed by a linear gradient of 0 to 17.5% eluent B (acetonitrile/0.1% formic acid) over 13 min. The peptidoglycan fragments were observed to elute between 5 and 8 min. Masses of the predicted $[M+H]^+$, $[M+2H]^{2+}$, and $[M+3H]^{3+}$ ions for the expected muropeptide fragments were extracted from the resulting total ion chromatograms to generate the extracted ion chromatograms displayed in the text and supplemental figures. Relative product amounts within a sample were calculated by

integrating the product peaks and dividing by the total peak area. Mass spectrometry data was analyzed using Thermo FreeStyle 1.5.

Characterization of Reaction Products. Digested peptidoglycan products A-E were reported by our group in a prior publication and were characterized by MS/MS fragmentation as previously described.[3] Representative mass spectra of each species from LC-MS experiments are given in Figure S4.

Cyclization Timecourse Reactions. To assess muramic-δ-lactam cyclization over time, an 800 μL pool of peptidoglycan enriched in Mur was produced as described in the general procedure above. For *Bs*SwsB^{WT} reactions, material eluting off of the Ni-NTA column was digested with mutanolysin (32 U) at 37 ºC for 2 h. For *Bs*PdaA and *Bs*SwsB^{Mut3} the mutanolysin digestion was omitted. The reaction mixture was split into two 400 μL volumes and EDTA (10 mM) or $ZnCl_2$ (2 μM) added to one aliquot as required. A *Bs*SwsB or *Bs*PdaA variant (2 μM) was then added and the reaction incubated static at room temperature. At timepoints, a 50 μL aliquot was removed and quenched by addition of 250 μL methanol. Samples were stored at -20 ºC prior to analysis. Methanolic samples were dried in a centrifugal evaporator and the residue resuspended in 50 μL of water. Mutanolysin digestion and LC-MS analysis were conducted as described above.

ATP Concentration in Protein Stocks. To measure ATP concentrations, a luminescence-based coupled enzyme assay was used (CellTiter-Glo 2.0, Promega). A calibration curve was prepared by serially diluting adenosine 5'-triphosphate disodium salt hydrate in assay buffer (50 mM HEPES pH 7.5) and transferring 100 μL aliquots of the standard solutions to a 96-well microtiter plate (PerkinElmer OptiPlate 96-HS, gray). Apyrase was added at 20 U/mL and the plate incubated 10 min at room temperature. 100 μL of CellTiter-Glo 2.0 reagent was then added to each well, the solutions mixed by pipet, and the plate incubated at room temperate for 10 min. Luminescence was measured on a PerkinElmer EnSpire multimode plate reader. To assess ATP concentrations in purified proteins, frozen stocks were diluted in assay buffer to a final concentration of 2 μM and the resulting solutions heat-denatured at 95 ºC for 10 min. Luminescence was measured on 100 μL aliquots of the protein solution as above.

Quantification of Metal Concentrations in Protein Samples. Metal concentrations in 200 μM protein stocks were determined by ICP-MS at the Dartmouth College Trace Metals Analysis Core, which is supported by Dartmouth Cancer Center and NCI Cancer Center Support Grant 5P30 CA023108.

Purification of tetrasaccharide C. Tetrasaccharide C (see main text Figure 2) was obtained by scaling up our procedure for generating Mur-enriched polymer. *B. subtilis* Lipid II was extracted from 3 L of culture as reported,[1,3] and the entire purified Lipid II extract (approximately 1 μmol) reconstituted in 200 μL DMSO. A polymerization reaction of 2 mL total volume was assembled with the following conditions: 50 mM HEPES pH 7.5, 500 μM Lipid II, 2 mM $CaCl_2$, 0.2 μM SgtB, 10% DMSO (v/v). *Bs*CwlD (2 μM) was added and the mixture incubated at room temperature for 2 hours. *Cd*PdaA1 (2 μM) was then added and the reaction incubated at room temperature for

30 min. The reaction was quenched by addition of EDTA (10 mM). The resulting Mur-enriched polymer was digested with mutanolysin (40 U) for 2 hours at 37 ºC with shaking. The tetrasaccharide C product was then purified by HPLC.

HPLC was conducted on an Agilent LC system consisting of a 1290 Infinity II binary pump, a 1260 Infinity II diode array detector, and a 1260 Infinity II fraction collector. Products were separated on a Waters XBridge BEH Shield RP18 column (130 Å, 5 µm, 10 x 150 mm) using the following method: 5 mL/min eluent A (water/0.1% formic acid) for 3 min followed by a linear gradient of 0 to 20% eluent B (acetonitrile/0.1% formic acid) over 30 min. The two tetrasaccharide C anomers were observed to elute between 10 and 12 mins with residual product A partially co-eluting. Fractions containing the product were pooled and lyophilized to dryness. The resulting residue was reconstituted in 200 µL water and quantified by derivatization with fluorescamine in 400 mM borate pH 9.7 using L-alaninamide as a standard. Estimated yields for this procedure were low (~5 %), but enough product was obtained for ~20 small scale reactions, as below.

Cyclization Reactions with Purified Tetrasaccharide C. Reactions with purified tetrasaccharide C were assembled under the following conditions: 50 mM HEPES, pH 7.5, 20 µM tetrasaccharide C, 2 µM *Bs*PdaA or *Bs*SwsB variant, 10% DMSO (v/v). Reactions were incubated at room temperature for 1 h. Aqueous sodium borohydride (10 mg/mL, 50 µL) was added and the mixture incubated 30 min at room temperature. The solution pH was adjusted to ~4 by addition of 20% phosphoric acid and lyophilized to dryness. Products were analyzed by LC-MS as above.

SUPPLEMENTAL FIGURES AND TABLES

Table S1: UniProt accession codes for proteins.

Protein	Accession
Staphylococcus aureus SgtB†	Q93Q23
Bacillus subtilis CwlD	P50864
Bacillus subtilis PdaA	O34928
Bacillus subtilis SwsB*	P50850
Clostridioides difficile PdaA1	Q18BV2

† SgtB is also known as Mgt1
* SwsB was previously known as YlxY

Table S2: Bacterial strains and plasmids

Strain or plasmid	Description[a]	Reference
E. coli		
C43(DE3)	BL21(DE3) derivative for protein expression	5
Plasmids		
pET24b	IPTG-inducible protein expression vector; Kan^R	Novagen
pET28b(+)	IPTG-inducible protein expression vector; Kan^R	Novagen
pMgt1†	*S. aureus* SgtB-His_6 expression vector; Amp^R	6
pMW1008	*B. subtilis* His_6-CwlD[N27-E237] expression vector, Kan^R	3
pMW1016	*C. difficile* His_6-PdaA[S29-K242] expression vector, Kan^R	3
pMW1070	*B. subtilis* PdaA[V24-L263]-His_6 expression vector, Kan^R	3
pMW1159-9	*B. subtilis* PdaA[V24-L263]^{H222A}-His_6 expression vector, Kan^R	This study
pMW1295	*B. subtilis* SwsB[K45-K319]-His_6 expression vector, Kan^R	This study
pMW1304	*B. subtilis* SwsB[K45-K319]^{N137D}-His_6 expression vector, Kan^R	This study
pMW2022	*B. subtilis* SwsB[K45-K319]^{N137D,A226R}-His_6 expression vector, Kan^R	This study
pMW2024	*B. subtilis* SwsB[K45-K319]^{L135T,N137D,A226R}-His_6 expression vector, Kan^R	This study
pMW2067	*B. subtilis* SwsB[K45-K319]^{H282A}-His_6 expression vector, Kan^R	This study
pMW2086	*B. subtilis* SwsB[E115-K319]-His_6 expression vector, Kan^R	This study

[a] Abbreviations: Amp^R, ampicillin resistance; Kan^R, kanamycin resistance
† SgtB is also known as Mgt1.

Table S3: Oligonucleotide PCR primers

primer	Sequence (5'-3')	Mutation
oMW47	CTCGAGCACCACCACCAC	
oMW104	CATATGTATATCTCCTTCTTAAAGTTAAACAAAATTATTTCTAGAGG	
oMW129	TTACCTGCTTGCGACCGTATCGAGGGACAATGCAGAAGCGCTGG	$PdaA^{H222A}$
oMW130	ATGGCTCCCGGGTGCGCC	$PdaA^{H222A}$
oMW179	TTTTTTAATCGATGTGGCATGGG	$SwsB^{N137D}$
oMW180	GCCACCATCGGTTTG	$SwsB^{N137D}$
oMW181	AAGAAGGAGATATACATATGAAAGACCCGTTATATGAAG	
oMW182	TGGTGGTGGTGGTGCTCGAGCTTCAATAGTCTTGTTTCATC	
oMW192	AAAGTGGTTCCGCCCGCCAAGCG	$SwsB^{N137D,A226R}$
oMW193	GGCTTAACGCCGATC	$SwsB^{N137D,A226R}$
oMW195	ATCGGTTTGTCAGGATTTC	$SwsB^{L135T,N137D,A226R}$
oMW200	GGTGGCTTTTACCATCGATGTGG	$SwsB^{L135T,N137D,A226R}$
oMW201	GATTTTAATGGCGCCGACTGACCCTACG	$SwsB^{H282A}$
oMW202	ATGGCACCATTATGTATC	$SwsB^{H282A}$
oMW211	AGAAGGAGATATACATATGGAGTCACTTCAGCCTGAG	

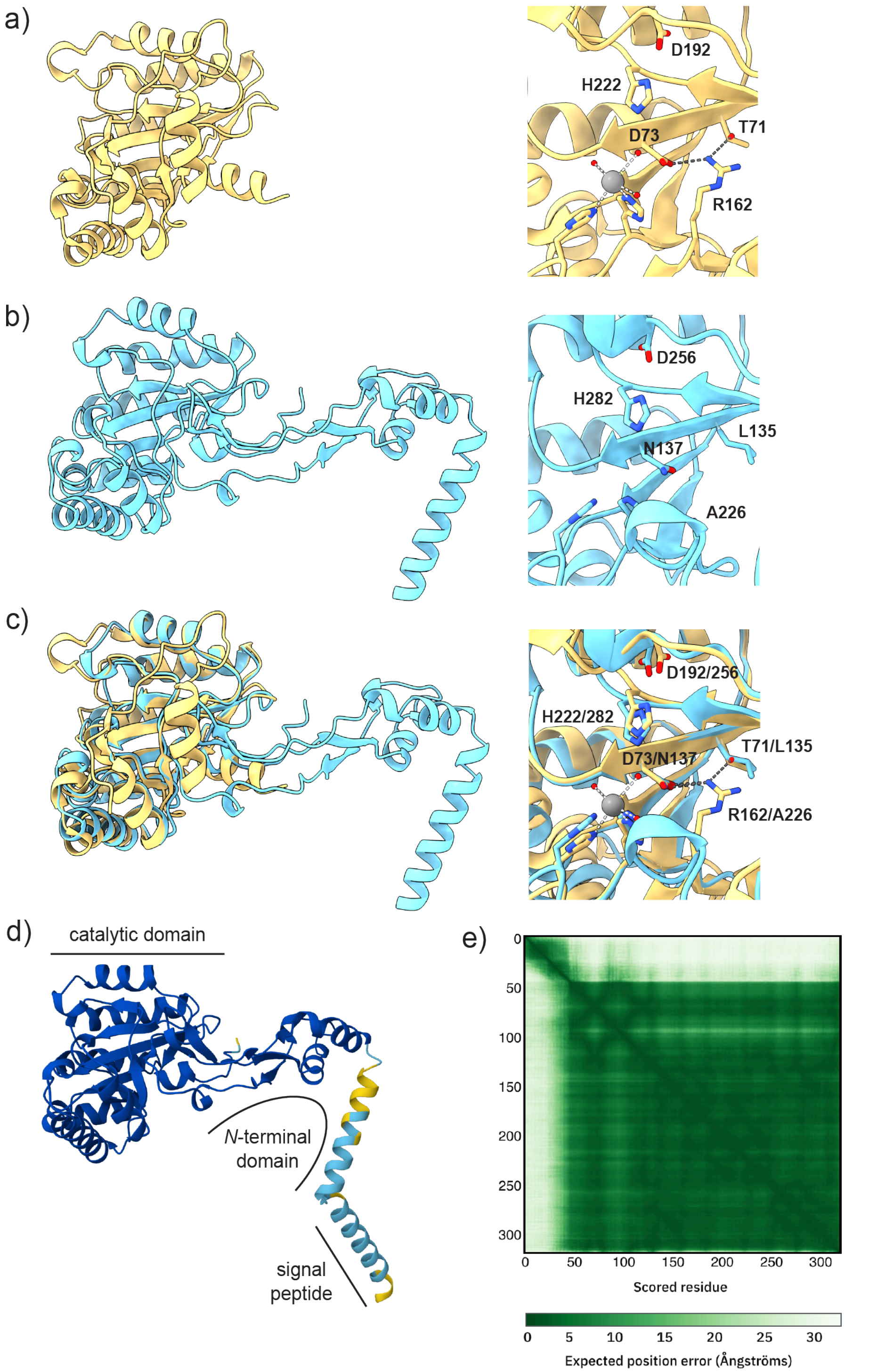

Figure S1. Structures of (a) *B. subtilis* PdaA (PDB 1W1B[7]) and (b) *B. subtilis* SwsB (AlphaFold3 model). The predicted *N*-terminal signal peptide is hidden in the SwsB structure. A bound divalent metal ion is shown as a gray sphere in the PdaA structure. Hydrogen bonds are shown as gray dashed lines. Coordinated water molecules are red spheres. (c) Structural alignment generated in ChimeraX version 1.9. (d) *Bs*SwsB AlphaFold3 model colored by residue confidence. Dark blue portions represent very high model confidence (pLDDT > 90). (e) AlphaFold3 model predicted alignment error.

```
Bs PdaA        ---MKWMCSICCAAVLLAG--GAAQ---AE-----------------------------A    23
Vh PdaA        ---MRNQVKILISILLFLSI---SL---PH-----------------------------T    22
Bc PdaA        -MKYKWLYMGLIFSIM--MALVPVS---AL-----------------------------A    25
Pm PdaA        ---MRRFTFFLVFLFVMSTGIASVE---AQ-----------------------------M    25
Bs SwsB        --MYKKFVPFAVFLFLFFVSFEMMENPHALDYIGAMKKDTVTVTASKDPLYEELLQKAPE    58
Vh SwsB        MYRLRQFITFCLFLLVVGISFNLDYNPFSEREGE----QLIQTTAKRDPLYQEILQKSSD    56
Bc SwsB        --MKVRILA-----------YICIFSLYVS--------LGSYSVFAQDNLHEEIQKHAKK    39
Pm SwsB        --MKRTIVQFTAFLFLLAITYKSIYNPFAEAYIEALKSDVQLVSAQHDALYQKIEEKAKD    58

Bs PdaA        VPNEPI------NWGFKRSVNHQPPDAGKQLNSLIE-----------------------K    54
Vh PdaA        ALAGGY------GWGYKKNNNHEIPDVGK-YKDMLD-----------------------K    52
Bc PdaA        YTNTPH------NWGIPRPKNETVPDAGKLYTDLLQ-----------------------K    56
Pm PdaA        YPNTPI------SWGFQKSKNHKPASAGTAYEQILA-----------------------K    56
Bs SwsB        YEVKPQNARIDKVWKSIPGYNGLKVNIEQSYKKMKQHGKFREKDLVYSQVKPSVHLESLQ    118
Vh SwsB        YAEEAQDAYIDKVWKKTPGRNGLQVNLEKSYRNMKESGEFDENLLSLEQTSPKISLEDLP    116
Bc SwsB        YEIAPQNAMIDKIWKATPGYNGRQVDIEASYNNMKKLKEFDQKYLEFKEVSPSVHLEDLS    99
Pm SwsB        YEKPAANARIDPVWKRVPGYNGIKVDLAASYKNMKPAGKFDEKKLVYKQVRPKVHLSDLP    118

Bs PdaA        YDAFYLGNTKEKTIYLTFDNGYENGYTPKVLDVLKKHRVTGTFFVTGHFVKDQPQLIKRM    114
Vh PdaA        YGAYYADFSGEKNIYLTFDNGYEEGYTDNILDVLKKEKVPATFFVTGHYVKDQPELVKRM    112
Bc PdaA        NGGFYLGDTKKKDIYLTFDNGYENGYTGKILDVLKEKKVPATFFVTGHYIKTQKDLLLRM    116
Pm PdaA        YDAFYLGDTNKKNIYLTFDNGYENGYTPQVLDVLKKRKVPAMFFVTGHYLKEEPKLIKRM    116
Bs SwsB        PEPIYKGNPDKPMVAFLINVAWGNEYLEKMLPILQKHQVKATFFLEGNWVRNNVQLAKKI    178
Vh SwsB        SAPIYRGHPEKEMVAFLINVSWGAEYIPDILNELKKEKVKATFFIEGKWAKENAELVKMI    176
Bc SwsB        PAPIYRGHPNKKMVGLTINVAWGNEYLPRILEILKKHDVKATFFLEGRWVKENLRFAKMI    159
Pm SwsB        QEPIYRGHDEKPMVSFTVNVAWGNEYLPKMLEVLKKHHAKATFFLEGKWVKNNPDMAKMI    178

Bs PdaA        SDEGHIIGNHSFHHPDLTTKTADQIQDELDSVNEEVYKITGKQDNLYLRPPRGVFSEYVL    174
Vh PdaA        VDEGHIIGNHSYHHPDFTIMDKDKIKKELQTLEKAVAEVSDQKSLRYVRPPRGTFSENTL    172
Bc PdaA        KDEGHIIGNHSWSHPDFTAVNDEKLREELTSVTEEIKKVTGQKEVKYVRPPRGVFSERTL    176
Pm PdaA        VKEGHIVGNHSWHHPDLTQVDDARFKEELQKVKDEYKNITGRDEMKYLRSPRGVFSERTL    176
Bs SwsB        AKDGHEIGNHSYNHPDMSKLTTGRISEQLDKTNEQIEQTIGVK-PKWFAPPSGSFRKAVI    237
Vh SwsB        SEQGHVIGNHAYNHPDMARLSNQKNIEQISSTNEIIKAIIDEE-PKWFAPPSGSYNQHVV    235
Bc SwsB        VDANQEVGNHSYTHPNMKTLSSDEIREQLQKTNRMIEVVTNQK-VRWFAPPSGSFRDEVV    218
Pm SwsB        VDAGHEVGNHSYSHPDMATLSASQINQQLKKTNDIITSTTGQK-VKWFAPPSGSFRPEVV    237

Bs PdaA        KETKRLGYQTVFWSVAFVDWKINNQKGKKYAYDHMIKQAHPGAIYLLHTVSRDNAEALDD    234
Vh PdaA        KWTYDLGYTHIFWSLAFIDWHTKKQKGWKYAYEQVMDQIHPGAIVLLHTVSSDNAEALQH    232
Bc PdaA        ALTKEMGYYNVFWSLAFLDWKVDQQRGWQYAHNNVMTMIHPGSILLLHAISKDNAEALAK    236
Pm PdaA        ALSKQEGYTNVFWSLAFVDWKVNEQKGWRYSYDNMMAQIHPGAIMLLHTVSKDNADALDQ    236
Bs SwsB        DIAAEKQMGTVMWTVDTIDWQKPAP---SVLQTRVLSKIHNGAMILMHPTDP-TAESLEA    293
Vh SwsB        DAAHNLNMHTILWTVDTIDWKNPTV---SVMINRVNDKLHPGATILMHPTES-IAEGIGP    291
Bc SwsB        KIADDFQMGTIMWTVDTIDWKRPEP---DVLLQRVMRKIHPGAIVLMHPTSS-TAEALDT    274
Pm SwsB        TLASQLKMSTIMWTVDTIDWQKPSS---EVLINRVMKKIHPGAIVLMHPTES-TAESLDQ    293

Bs PdaA        AITDLKKQGYTFKSIDDLMFEKEMRLPSL----      263
Vh PdaA        MITELKKQGYSFKSLDELVMKDHIPKVIYGLE-      264
Bc PdaA        IIDDLREKGYHFKSLDDLVKGNQP---------      260
Pm PdaA        AIVDLKKQGYTFKSIDDLMNERKQMKKSPSETK      269
Bs SwsB        LITQIKDKGYALGTVTELMDETRLLK-------      319
Vh SwsB        LIRQVKQKGFKLGTIEKLLNEER----------      314
Bc SwsB        MIKKLKEQGYKVGNITELLDEKRVD--------      299
Pm SwsB        LLTDIERKGLKVSDVSTMLDEERMMKIPSSTKK      326
```

text = conserved structural or substrate-/cofactor-binding residues
text = semi-conserved or complementary structural residue
text = catalytic or active site residue, conserved in both proteins
text = catalytic or active site residue, conserved in PdaA variants
text = catalytic or active site residue, conserved in SwsB variants

Figure S2. Multiple protein alignment of selected PdaA and SwsB variants. Sequences were obtained from the KEGG genome database (https://www.genome.jp/kegg/genome) and aligned using NCBI Protein BLAST. Species labels are *Bs* – *Bacillus subtilis* 168, *Vh* – *Virgibacillus halodenitificans* PDB-F2, *Bc* – *Bacillus cereus* ATCC 14579, *Pm* – *Priestia megaterium* QM B1551. The UniProt entries for each protein are as follows: *Bs* PdaA – O34928, *Bs* SwsB – P50850, *Vh* PdaA – A0AAC9IXT6, *Vh* SwsB – A0AAC9J0E3, *Bc* PdaA – Q81IC4, *Bc* SwsB – Q819Z2, *Pm* PdaA – D5DXQ5, *Pm* SwsB – D5DQ55.

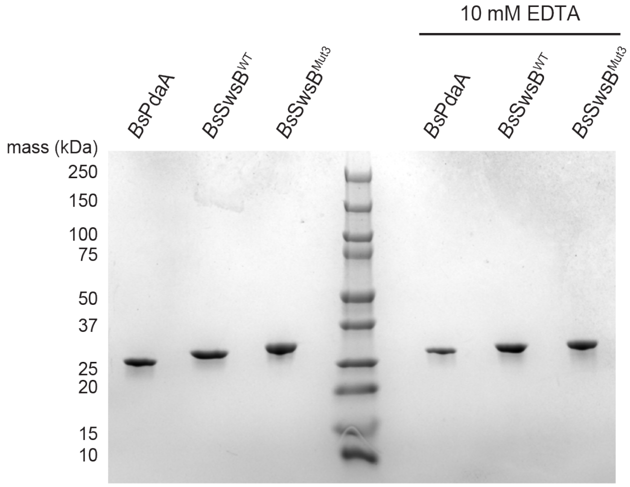

Figure S3. Coomassie-stained SDS-PAGE of purified proteins. Proteins in the "10 mM EDTA" lanes were purified according to the metal-stripping protocol (see Methods). Protein was loaded at approximately 2 µg per lane.

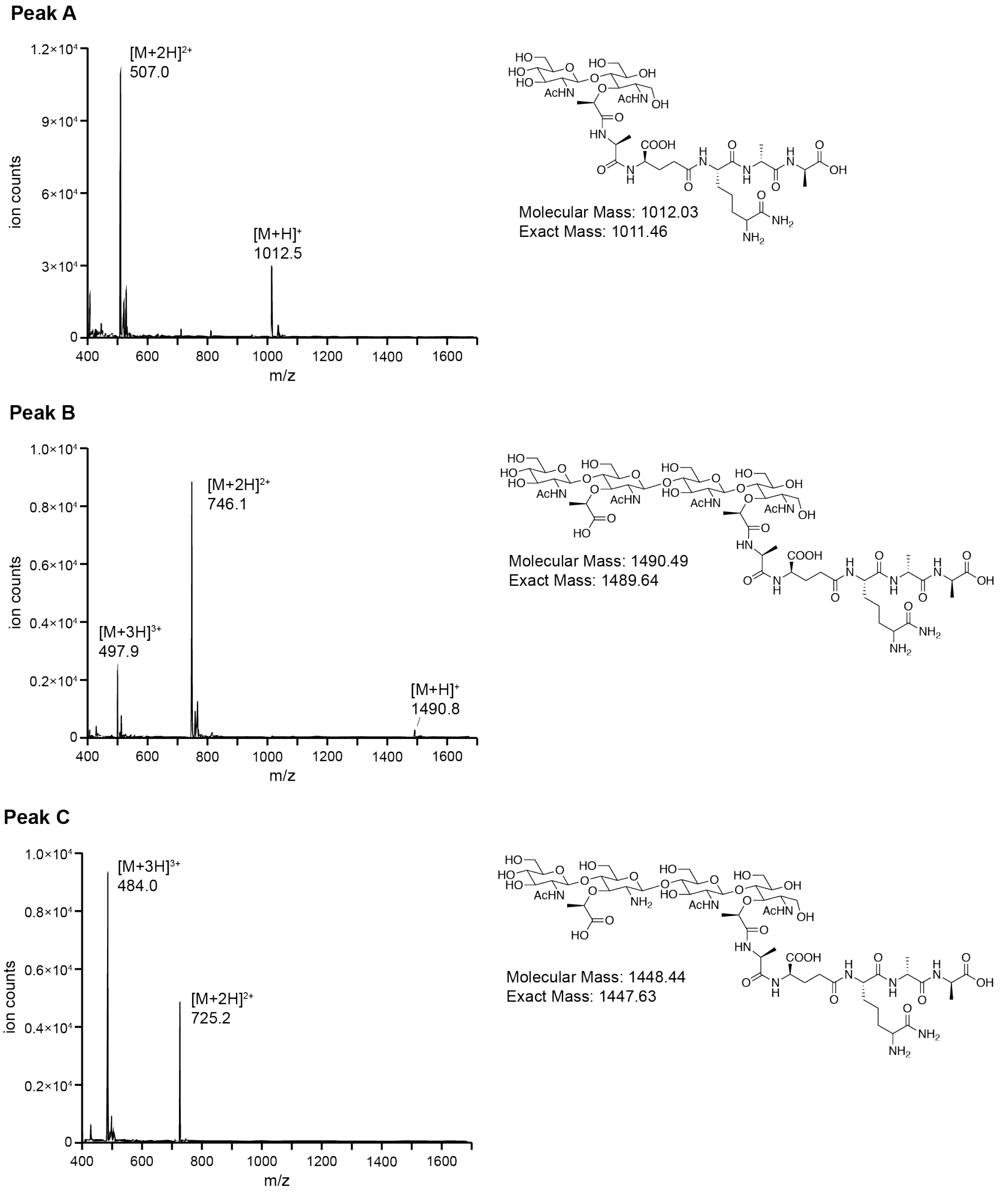

Figure S4. Electrospray ionization mass spectra of peptidoglycan fragments. Products A-E are defined as in the main text. The diagnostic ion adducts are labeled for each species.

Peak D

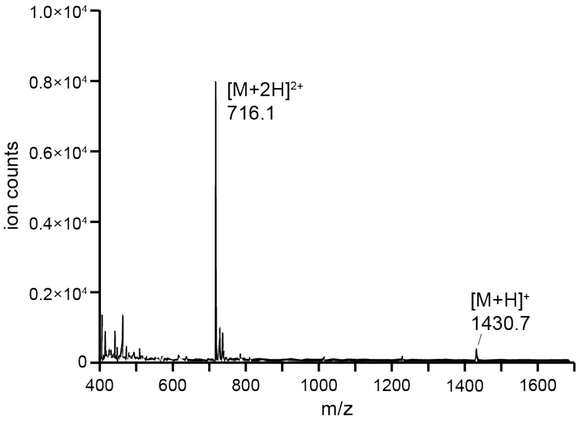

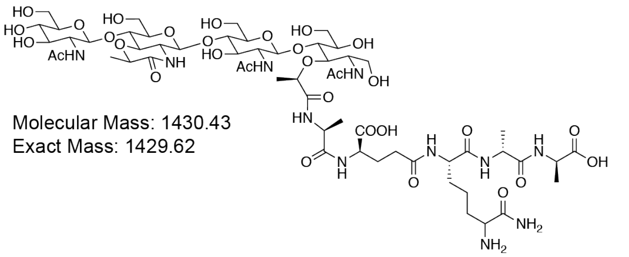

Peak E

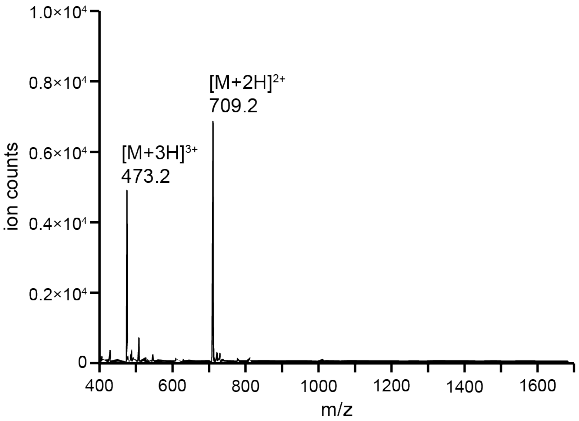

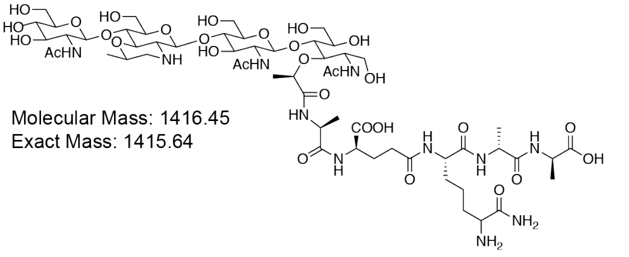

Figure S4 (continued). Electrospray ionization mass spectra of peptidoglycan fragments. Products A-E are defined as in the main text. The diagnostic ion adducts are labeled for each species.

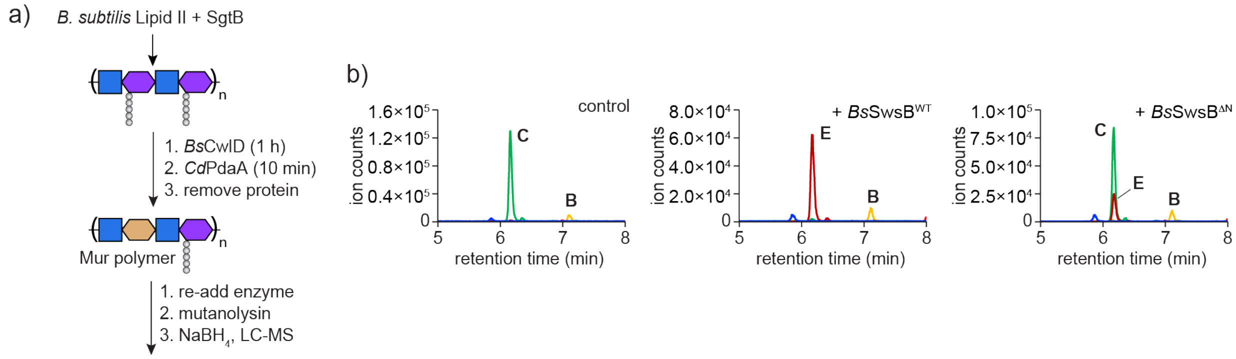

Figure S5. The SwsB *N*-terminal domain is not required for cyclase activity. a) Schematic of the experimental workflow. Enzymes were re-added to peptidoglycan enriched in Mur and incubated for 2 h. Polymer products were digested with mutanolysin and analyzed by LC-MS. b) LC-MS extracted ion chromatograms of an untreated control and reactions with *Bs*SwsB^{WT} or *Bs*SwsB$^{\Delta N}$. Data are representative of two independent experiments.

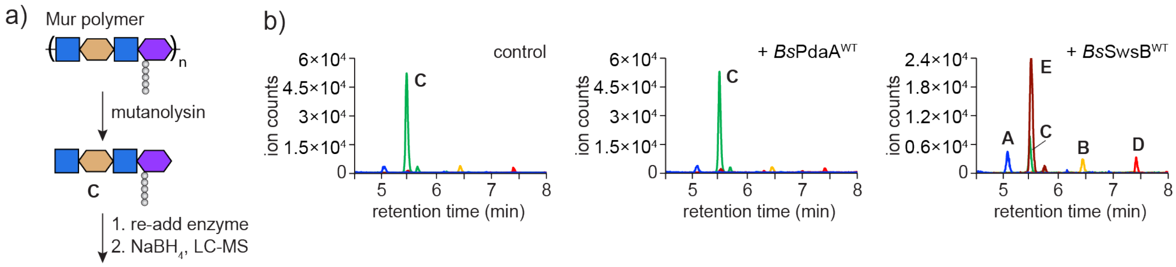

Figure S6. *Bs*SwsB will accept digested peptidoglycan as a substrate. a) Schematic of experimental workflow. Peptidoglycan enriched in Mur was digested with mutanolysin to produce tetrasaccharide C. Enzymes were re-added to the digested material for 1 h and the reaction products analyzed by LC-MS. b) LC-MS extracted ion chromatograms of an untreated control and reactions with *Bs*PdaA or *Bs*SwsB. Data are representative of two independent experiments.

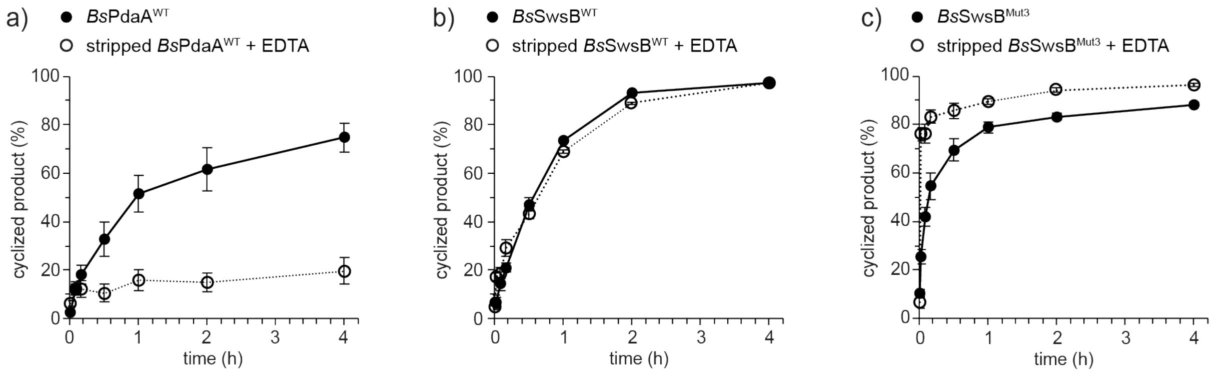

Figure S7. Analysis of muramic-δ-lactam synthesis over time. Pooled reactions were assembled using Mur polymer as a substrate for (a) *Bs*PdaA and (c) *Bs*SwsB^{Mut3} or digested tetrasaccharide C for (b) *Bs*SwsB^{WT}. Open circles are reactions with metal-stripped protein (2 μM) in the presence of 10 mM EDTA. At timepoints, aliquots were removed, methanol-quenched, and the products were analyzed by LC-MS. Error bars represent the standard error of three independent experiments. Data in panels a and b is reproduced from Figure 3a.

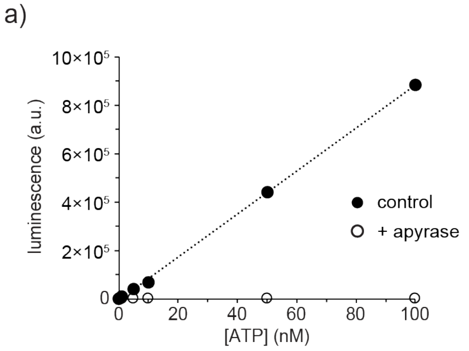

b)

	[ATP] (nM)	
heat denatured	−	+
*Bs*PdaA	< 1	< 1
*Cd*PdaA	< 1	< 1
*Bs*SwsB	< 1	< 1
*Bs*SwsB^{Mut3}	< 1	< 1

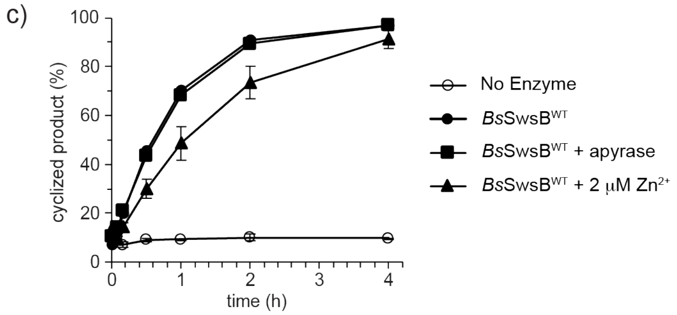

Figure S8. SwsB-catalyzed lactam synthesis is unaffected by apyrase and inhibited by Zn^{2+}. (a) Calibration curve for luminescence-based ATP detection (CellTiter-Glo 2.0, Promega). ATP standards were incubated with apyrase (20 U/mL) for 10 min at room temperature. (b) Maximum ATP concentrations detected in purified protein stocks. Protein samples were assayed untreated or after heating at 95 ºC for 10 min to release bound cofactors. In all samples, luminescence signal was below the limit of detection of the assay. (c) Cyclization reactions with *Bs*SwsB^{WT} (2 μM) and digested tetrasaccharide C. *Bs*SwsB^{WT} was pre-incubated with apyrase (20 U/mL) or $ZnCl_2$ (2 μM) for 10 min at room temperature before addition to the reaction mixture. At timepoints, aliquots were removed, methanol-quenched, and the products were analyzed by LC-MS. Error bars represent the standard error of three independent experiments.

	Concentration (μM)							
**Sample**	**Mn**	**Fe**	**Co**	**Ni**	**Cu**	**Zn**	**Total**	**Mol%**
Buffer	0.03	1.21	0.00	0.03	0.03	0.19	1.50 ± 1.4	
*Cd*PdaA	0.14	0.98	0.01	9.25	1.09	16.64	28.09	14
*Bs*CwlD	1.36	0.45	0.16	36.12	3.98	49.91	91.98	46
*Bs*PdaA	0.78	0.93	0.01	3.81	1.29	4.95	11.78 ± 2.6	6 ± 1.3
*Bs*SwsB^{WT}	0.03	1.09	0.00	1.11	0.15	1.95	4.33 ± 0.1	2 ± 0.1
*Bs*SwsB^{Mut3}	0.01	0.36	0.00	1.81	0.12	1.39	3.69	2

Table S4. Transition metal concentrations in purified protein samples. Protein stocks were submitted for ICP-MS analysis at the Dartmouth College Trace Metal Analysis Core. Means and the standard error of 2-3 replicates are reported for the buffer control, *Bs*PdaA, and *Bs*SwsB. Buffer is 50 mM HEPES, 400 mM NaCl, pH 7.5. Mol% was determined relative to the protein stock concentration, 200 μM.

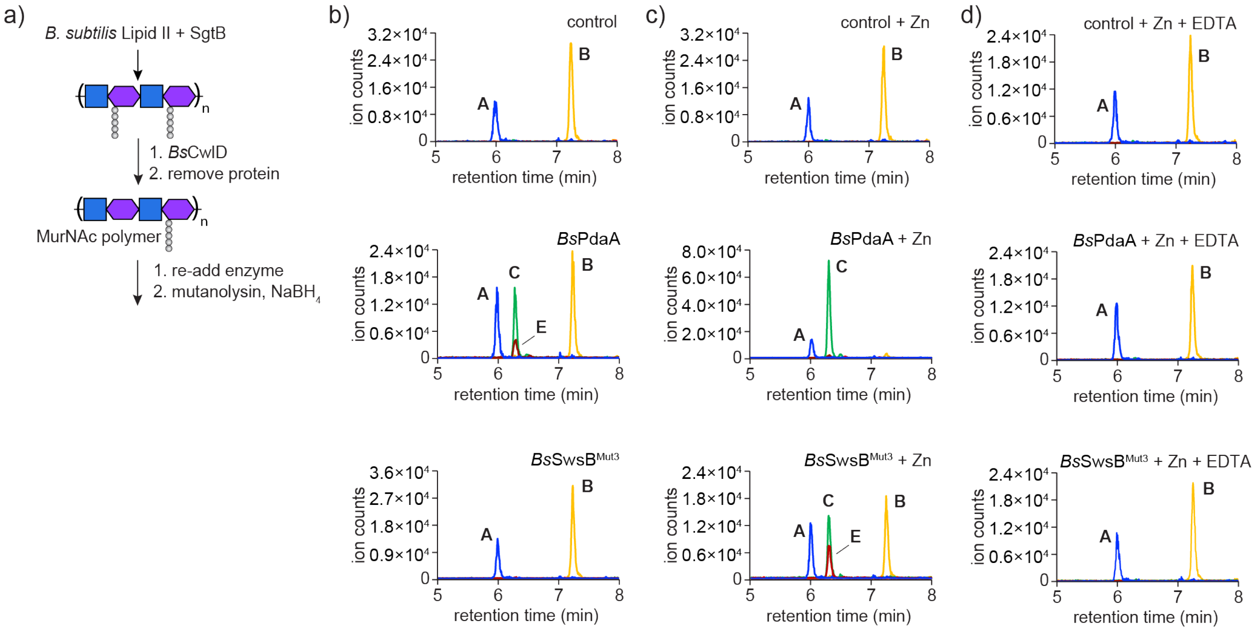

Figure S9. Deacetylase activity can be recovered in the metal-stripped proteins by addition of Zn^{2+}. (a) Schematic of experimental workflow. Linear peptidoglycan was incubated with *Bs*CwlD for 1 h to produce polymer enriched in peptide-cleaved MurNAc. Metal-stripped *Bs*PdaA or *Bs*SwsB^{Mut3} (2 μM) were added to reactions supplemented with (b) no additive, (c) 110 μM $ZnCl_2$, or (d) 110 μM $ZnCl_2$ + 10 mM EDTA (d). The 110 μM $ZnCl_2$ concentration was chosen because the metal-stripped protein aliquots contain EDTA themselves (see Methods), giving a final EDTA concentration of 100 μM upon dilution into the reaction mixture. Reactions were incubated 1 h at room temperature and the products analyzed by LC-MS. The data are representative of two independent experiments.

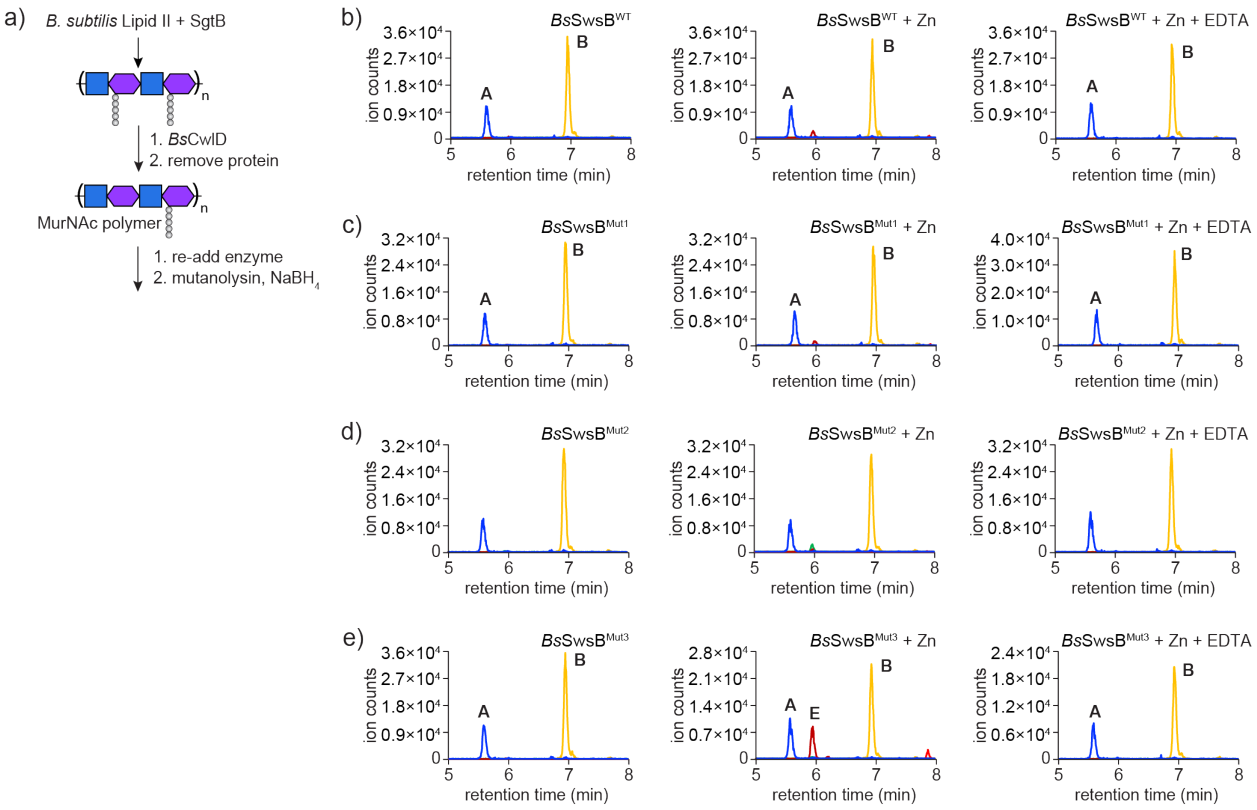

Figure S10. Generation of a SwsB variant with deacetylase activity. (a) Schematic of experimental workflow. Linear peptidoglycan was incubated with *Bs*CwlD for 1 h to produce polymer enriched in peptide-cleaved MurNAc. Polymer aliquots were supplemented with 2 µM $ZnCl_2$ or 2 µM $ZnCl_2$ + 5 mM EDTA. Purified SwsB variants were added at 2 µM and the reactions incubated at room temperature for 3 h. The variants are as follows: (b) wild-type *Bs*SwsB; (c) *Bs*SwsB^{Mut1} = N137D; (d) *Bs*SwsB^{Mut2} = N137D,A226R; (e) *Bs*SwsB^{Mut3} = N137D,A226R,L135T. Data are representative of three independent experiments.

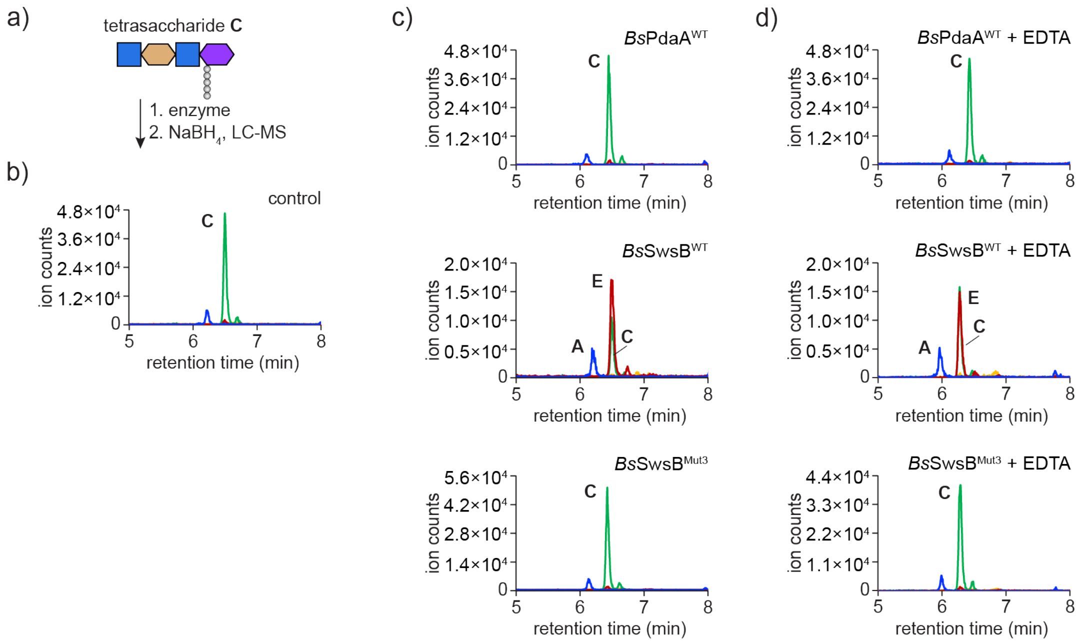

Figure S11. Reactions with purified tetrasaccharide C. (a) Schematic of experimental workflow. See Methods for the experimental protocol to produce purified C. Tetrasaccharide C (20 μM) and the indicated enzymes (2 μM) were incubated in 50 mM HEPES pH 7.5 at room temperature for 1 h and the products analyzed by LC-MS. (b) No enzyme control. (c) Reactions without EDTA in the reaction buffer. (d) Reactions of metal-stripped enzymes in buffer supplemented with 10 mM EDTA. The data are representative of two independent experiments.

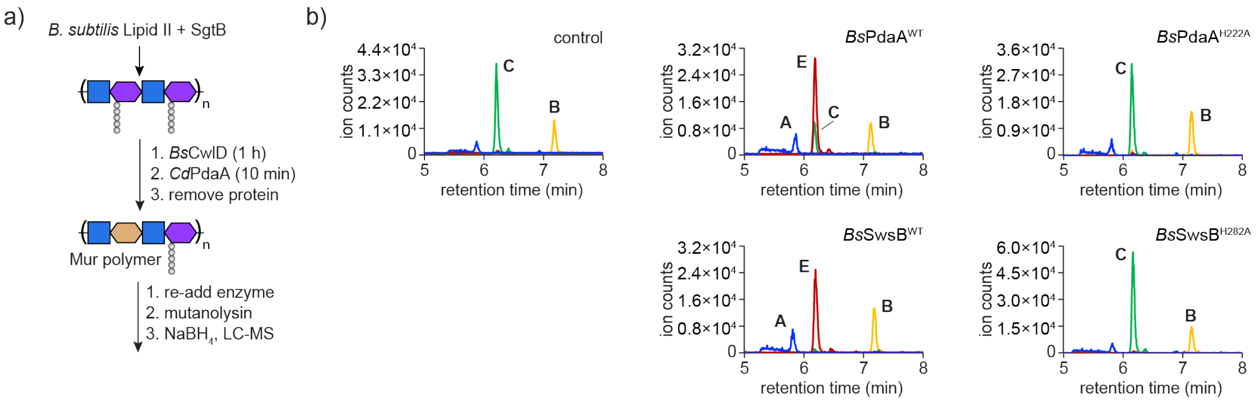

Figure S12. Mutation of an invariant active site His abolishes cyclase activity. (a) Schematic of experimental workflow. Enzymes (2 μM) were incubated with Mur-enriched polymer for 1 h at room temperature. (b) LC-MS extracted ion chromatograms of cyclization reaction products. The data are representative of two independent experiments.

References

(1) Qiao, Y.; Srisuknimit, V.; Rubino, F.; Schaefer, K.; Ruiz, N.; Walker, S.; Kahne, D. Lipid II Overproduction Allows Direct Assay of Transpeptidase Inhibition by β-Lactams. *Nat. Chem. Biol.* **2017**, *13* (7), 793–798. https://doi.org/10.1038/nchembio.2388.

(2) Rebets, Y.; Lupoli, T.; Qiao, Y.; Schirner, K.; Villet, R.; Hooper, D.; Kahne, D.; Walker, S. Moenomycin Resistance Mutations in *Staphylococcus aureus* Reduce Peptidoglycan Chain Length and Cause Aberrant Cell Division. *ACS Chem. Biol.* **2014**, *9* (2), 459–467. https://doi.org/10.1021/cb4006744.

(3) Tobin, M. J.; Cho, S. Y.; Profy, W.; Ryan, T. M.; Le, D. H.; Lin, C.; Yip, E. Z.; Dorsey, J. L.; Levy, B. R.; Rhodes, J. D.; Welsh, M. A. Reconstituting Spore Cortex Peptidoglycan Biosynthesis Reveals a Deacetylase That Catalyzes Transamidation. *Biochemistry* **2023**, *62* (8), 1342–1346. https://doi.org/10.1021/acs.biochem.3c00100.

(4) Gasteiger, E.; Hoogland, C.; Gattiker, A.; Duvaud, S.; Wilkins, M. R.; Appel, R. D.; Bairoch, A. Protein Identification and Analysis Tools on the Expasy Server. In *The Proteomics Protocols Handbook*; Walker, J. M., Ed.; Humana Press, 2005; pp 571–607. https://doi.org/10.1385/1-59259-890-0:571.

(5) Miroux, B.; Walker, J. E. Over-Production of Proteins in *Escherichia coli*: Mutant Hosts That Allow Synthesis of Some Membrane Proteins and Globular Proteins at High Levels. *J. Mol. Biol.* **1996**, *260* (3), 289–298. https://doi.org/10.1006/jmbi.1996.0399.

(6) Heaslet, H.; Shaw, B.; Mistry, A.; Miller, A. A. Characterization of the Active Site of *S. aureus* Monofunctional Glycosyltransferase (Mtg) by Site-Directed Mutation and Structural Analysis of the Protein Complexed with Moenomycin. *J. Struct. Biol.* **2009**, *167* (2), 129–135. https://doi.org/10.1016/j.jsb.2009.04.010.

(7) Blair, D. E.; van Aalten, D. M. F. Structures of *Bacillus subtilis* PdaA, a Family 4 Carbohydrate Esterase, and a Complex with N-Acetyl-Glucosamine. *FEBS Lett.* **2004**, *570* (1–3), 13–19. https://doi.org/10.1016/j.febslet.2004.06.013.
